# Covalent Organic Frameworks Based Electrocatalysts for Two-Electron Oxygen Reduction Reaction: Design Principles, Recent Advances, and Perspective

**DOI:** 10.3390/molecules29112563

**Published:** 2024-05-30

**Authors:** Rui Qiao, Jinyan Wang, Hongyin Hu, Shuanglong Lu

**Affiliations:** Key Laboratory of Synthetic and Biological Colloids, Ministry of Education, School of Chemical and Material Engineering, Jiangnan University, Wuxi 214122, China

**Keywords:** COFs, electrocatalytic, 2e^−^ ORR, H_2_O_2_

## Abstract

Hydrogen peroxide (H_2_O_2_) is an environmentally friendly oxidant with a wide range of applications, and the two-electron pathway (2e^−^) of the oxygen reduction reaction (ORR) for H_2_O_2_ production has attracted much attention due to its eco-friendly nature and operational simplicity in contrast to the conventional anthraquinone process. The challenge is to design electrocatalysts with high activity and selectivity and to understand their structure–activity relationship and catalytic mechanism in the ORR process. Covalent organic frameworks (COFs) provide an efficient template for the construction of highly efficient electrocatalysts due to their designable structure, excellent stability, and controllable porosity. This review firstly outlines the design principles of COFs, including the selection of metallic and nonmetallic active sites, the modulation of the electronic structure of the active sites, and the dimensionality modulation of the COFs, to provide guidance for improving the production performance of H_2_O_2_. Subsequently, representative results are summarized in terms of both metallic and metal-free sites to follow the latest progress. Moreover, the challenges and perspectives of 2e^−^ ORR electrocatalysts based on COFs are discussed.

## 1. Introduction

Hydrogen peroxide (H_2_O_2_) is widely used as an environment-friendly oxidizing agent in medical, agricultural, and industrial applications, which plays a pivotal role in environmental purification, chemical synthesis, and textile bleaching [1,2,3,4]. It is predicted that the demand for H_2_O_2_ is still burgeoning, expected to reach 5.7 million metric tons by 2027 [5]. However, the traditional H_2_O_2_ production mainly relies on the anthraquinone process, which requires expensive and large equipment and infrastructure support. Moreover, the process also produces a large number of organic by-products, which has a negative impact on the environment [5,6,7]. Another method is the direct synthesis of H_2_O_2_ by the reaction of H_2_ and O_2_. However, the yield will be severely reduced due to the fact that H_2_ is easily oxidized to H_2_O instead of H_2_O_2_. Additionally, the combustible nature of H_2_/O_2_ mixtures presents a notable safety hazard, which somewhat hinders the practical feasibility of this method [8]. Consequently, it presents a need to explore more efficient and cost-effective avenues for H_2_O_2_ production.

The in situ synthesis of H_2_O_2_ can be achieved directly by electrocatalytic oxygen reduction reaction (ORR), utilizing oxygen and water as feedstock [9,10,11]. This method is characterized by low cost, high efficiency, safety, and timeliness, which makes it a promising alternative to the traditional anthraquinone process [12]. Due to the thermodynamic preference of ORR towards the four-electron pathway, adjusting the absorption behavior of reactants and intermediates to achieve the efficient synthesis of H_2_O_2_ via the two-electron ORR presents a standing challenge [13,14]. In order to overcome this challenge, various electrocatalysts have been developed, including precious metal-based, transition metal-based, and metal-free electrocatalysts [15]. Among them, precious metal-based catalysts such as platinum [16] and palladium [17] exhibit high catalytic activity, but their limited reserves and high cost significantly restrict their widespread application [18,19], while transition metal-based catalysts with relatively low cost suffer from insufficient activity and relatively poor long-term stability. Metal-free based catalysts like carbon-based catalysts have garnered attention due to their low cost, abundant resources, and eco-friendliness [20,21]. However, existing carbon-based catalysts are predominantly prepared through post-modification or pyrolysis of porous polymers, leading to their uncontrollable porous structures and disordered active sites. Furthermore, the unclear structure of carbon materials doped with heteroatoms hinders the understanding of reaction mechanisms and structure-activity relationships, thus impacting the selectivity and efficiency of H_2_O_2_ generation [22]. Considering these progresses made and problems faced, the development of efficient and structurally defined electrocatalysts is still highly needed.

Covalent organic frameworks (COFs) have emerged as novel porous crystalline polymers distinguished by their periodic framework structures and ordered pores [23,24,25,26]. Typically, they possess precisely controllable monomers, large specific surface area, and high stability, which make them an efficient platform for electrocatalysis [27,28,29]. Specifically, the remarkable advantages of COFs have received widespread attention in electrocatalytic ORR [30,31,32,33,34,35,36]. The large specific surface area and porous nature of COFs can provide sufficient active sites to participate in the catalytic reaction [37]. Moreover, the inherent porous structure and conjugated organic structure are favorable for ion and charge carrier transport, which is conductive to the enhancement of the reaction rate. More importantly, COFs exhibit a robust and easy-to-functionalize framework structure, which provides a superior platform for realizing COF-based composites and derived materials with advantageous properties [38,39]. This property also provides an advantage in understanding the relationship between structure and activity during the ORR process, providing a basis for the further optimization of their activity and selectivity. Therefore, the use of structure-specific COFs platforms brings new opportunities to design efficient and cost-effective catalysts for targeted 2e^−^ ORR.

**Figure 1:**
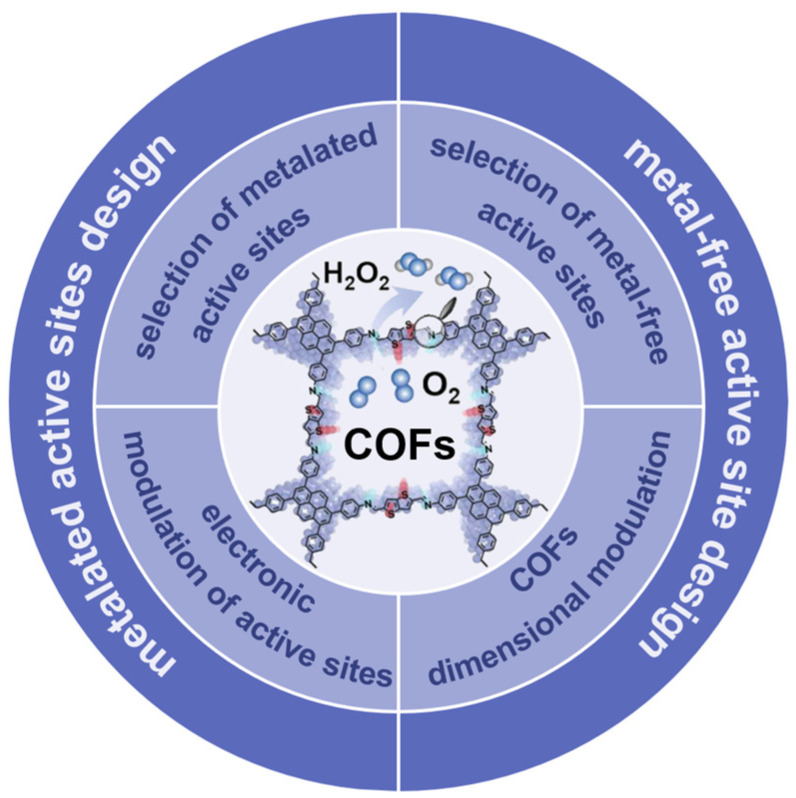
Classification and design principles of COFs for 2e^−^ ORR.

This review first introduces the design principles, including the selection of metallic and metal-free active sites, the modulation of the electronic structure of the active sites, and the dimensionality modulation of the COFs, to provide guidance for the optimization of COFs toward the improvement of 2e^−^ ORR (Figure 1). Representative results in this field have been promptly summarized, offering insights for further development and utilization of COF materials for electrochemical applications. Finally, the perspective and challenges for the development of COF-based 2e^−^ ORR electrocatalysts are presented.

## 2. Introduction to Electrocatalytic ORR

**Figure 2:**
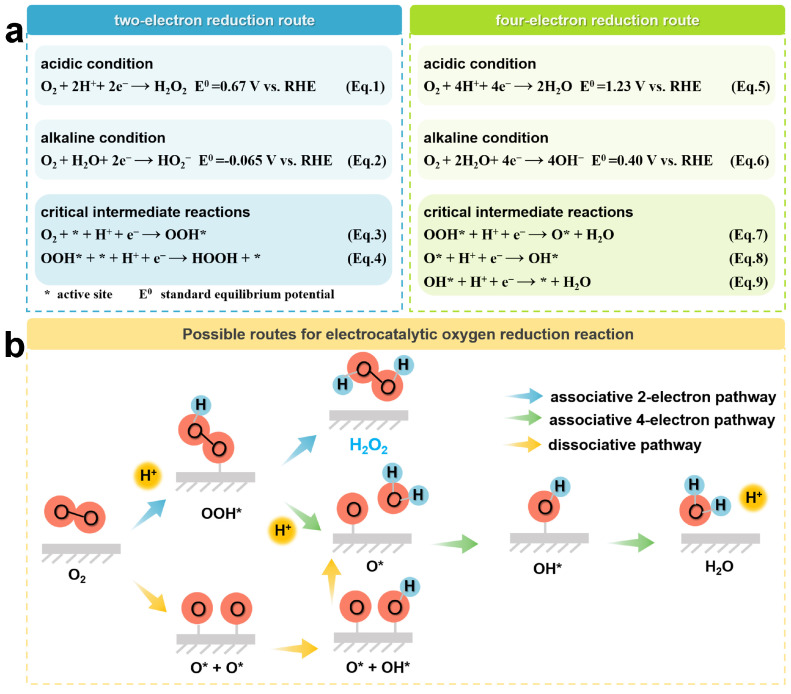
(**a**) Potentiometric equations and (**b**) Possible pathways for the ORR process.

Electrocatalytic ORR is an electrochemical reaction occurring at the gas–liquid–solid three-phase interface, which mainly involves the processes of adsorption of O_2_ molecules, proton and electron transfer, bond breaking or formation, and product release [40,41,42]. Typically, the ORR process can generate H_2_O_2_ via the 2e-transfer pathway, as shown in Equations (1) and (2), or generate H_2_O via the 4e-transfer pathway, as shown in Equations (5) and (6) [43]. In these equations, RHE stands for the reversible hydrogen electrode. The further detailed elucidation of the adsorption behavior of different intermediates on the catalyst during the ORR process is shown in Figure 2a. Ideally, the catalyst should possess a high adsorption capacity for O_2_ molecules and a weak adsorption capacity for peroxyl radicals (OOH*), which is favorable for the generation of OOH* and the release of products, respectively [44]. In order to develop catalysts with high reactivity, the energy barriers for O_2_ molecular activation and OOH* desorption need to be minimized. OOH* is the key intermediate in 2e^−^ ORR. Therefore, the Gibbs free energy of OOH* (ΔG_OOH*_) is considered as a descriptor to predict the catalyst activity [45,46,47,48]. In general, the optimal ΔG_OOH*_ applicable to 2e^−^ ORR should be close to the peak of the volcano diagram [47]. Typically, OOH* may undergo further reduction to complete the 4e^−^ pathway. It is thermodynamically unfavorable for 2e^−^ ORR, and the resulting H_2_O_2_ is likely to be further reduced to H_2_O. In order to maintain the 2e^−^ pathway, the O–O bonds between the O_2_ molecules have to be preserved, and a strong interaction between the active site and oxygen should be avoided. Otherwise, the O–O bonds tend to dissociate, eventually leading to the formation of H_2_O. In this scenario, the O* produced from the dissociation of OOH* is only weakly bound to the reaction site. However, weakening the binding strength of O* will also decrease the binding ability of OOH*, which ultimately lowers the degree of activation of oxygen molecules. This relationship arises from the proportionality in free energies among oxygenated intermediates (OOH*, O*, and OH*) [44,49]. Therefore, targeted modulation of the binding strength between oxygen-containing intermediates is an effective strategy to simultaneously achieve high reactivity and selective H_2_O_2_ production from the catalysts.

The 2e^−^ ORR electrochemical tests are mainly performed using a standard three-electrode system with the aid of a rotating ring disk electrode (RRDE). The analysis of electrocatalytic ORR performance and selectivity of catalysts are mainly based on tests such as linear scanning voltammetry (LSV), cyclic voltammetry (CV), chronoamperometry (CA), and electrochemical impedance spectroscopy (EIS). By analyzing the electrochemical profiles and quantitative results, several basic parameters could be obtained, which are commonly used to evaluate the selectivity and activity of electrocatalysts toward 2e^−^ ORR. These parameters include the onset potential (E_0_), half-wave potential (E_1/2_), number of transferred electrons (n), Tafel slope, limiting diffusion current density (J_lim_), Faraday efficiency (FE), and turnover frequency (TOF). Among them, the number of transferred electrons is a marker for electron transfer pathways, FE indicates energy utilization efficiency, and TOF reflects the intrinsic activity of the catalyst. Their calculation formulas are as follows:

n = 4I_D_/(I_D_ + I_R_/N)

FE = 100 I_R_/(N × I_D_)

TOF = J_K_ × FE × S_A_/n × N × F

In this context, I_D_ and I_R_ are the currents collected by the disk electrode and the ring electrode, respectively, N is the collection coefficient, J_K_ is the kinetic current density, S_A_ is the geometric surface area of the disk electrode, and F is the Faraday constant.

## 3. Design Principles for COFs with High Electrocatalytic 2e^−^ ORR Performance

COF materials have received much attention in electrocatalytic ORR due to their structural diversity, customizability, excellent stability, and unique pore characteristics. Utilizing COF materials with specific structures to design efficient and low-cost catalysts for directed electrocatalytic ORR has become the research focus. In this section, we will propose several design principles of COFs toward two-electron ORR, including the selection of metal-free active sites, the selection of metalated active sites, the electronic modulation of active sites, and the dimensional modulation of organic framework, to reveal the possible factors affecting the electrocatalytic performance of COFs.

**Figure 3:**
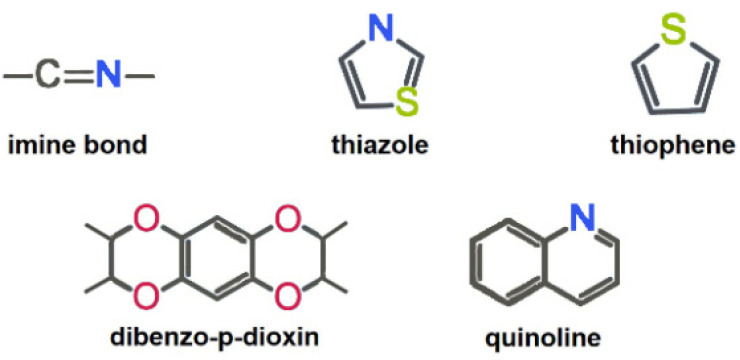
Typical nonmetallic active units of COFs for 2e^−^ ORR.

**a. Selection of metal-free active sites.** The introduction of heteroatoms is considered as an important strategy to induce metal-free electrocatalytic active sites in the structure of COFs [21,22]. Heteroatoms can effectively regulate the charge distribution of adjacent carbon atoms, thereby significantly influencing the catalytic activity, selectivity, and stability of COFs [14]. The electronic modulation of COFs could enhance the adsorption and activation of oxygen-containing intermediates in the electrocatalytic ORR process [34]. By fully utilizing the electronegativity, chemical bonding state and atomic size differences between doped atoms and carbon atoms, metal-free active sites could be induced on the COF backbone. Typical previously reported metal-free active sites toward 2e^−^ ORR are shown in Figure 3.

**b. Selection of metalated active sites.** Due to the highly customizable nature of the building blocks, the selection of suitable ligands (e.g., porphyrins, bipyridines, and phthalocyanines) to chelate metal species in the backbone of the COFs for catalytic ORR is also a common strategy [50,51]. These building blocks are usually rich in heteroatoms whose lone-pair electrons can be coordinated to the metal, and the metal is doped into the COFs to form a metallic active site through controlled bonding. Generally, metalated active sites are responsible for exhibiting different affinities toward O_2_ molecules and displaying diverse binding energies of adsorption intermediates during the proton–electron transfer processes. In addition, the electronic structure of the active site can be effectively modulated by the selection of the metal center, which enables the molecular level modulation of the electronic environment of the active center to influence the ORR activity of the catalyst. Previously reported coordinated metal species involve Mg, Ca, Mn, Fe, Co, Ni, Cu, and Zn [52,53,54,55,56].

**c. Electronic modulation of active sites.** The adsorption behavior of O_2_ molecules relies heavily on the geometry and electronic structure of the active site. A greater disparity in charge density between the active site and the O_2_ molecule enhances the rate of electron transfer from the catalyst to the O_2_ molecule. This acceleration not only assists in O_2_ adsorption during the ORR process but also facilitates subsequent reactions of the adsorbed O_2_ molecules [40]. According to previous reports, the introduction of heteroatoms with different electronegativities, the surface modification with functional groups of varying electron affinities, the adjustment of linkage bonds and structural units, chelated metal ions, and axial coordination of metal centers can all be used for electronic regulation of active sites.

**d. COF dimensional modulation.** The structural design of COF materials is based on topology. COFs can be categorized into one-dimensional COFs (1D COFs), two-dimensional COFs (2D COFs) with a layered structure, and three-dimensional COFs (3D COFs) with a reticular structure. Compared to 2D COFs, 1D COFs have more edge sites and lower density of basal sites, while 3D COFs are of higher dimensionality with larger porosity and specific surface area. However, due to the limited number of reports on one-dimensional and three-dimensional COFs, it is challenging to draw definitive conclusions through direct comparisons.

## 4. COFs for Electrocatalytic H_2_O_2_ Production

COFs have the advantages of high specific surface area, high porosity, and designable backbone structure, which make them efficient for tailoring efficient electrocatalytic 2e^−^ ORR catalysts. How to utilize the structural advantages of COFs to modulate their structural composition and electronic properties to enhance their electrocatalytic activity has become a hot research topic. To date, different strategies have been used to modulate the electrocatalytic ORR performance and selectivity of COF materials. In this section, we will focus on the design of non-metallic active sites and metallic active sites to illustrate the research progress of COF materials in electrocatalytic 2e^−^ ORR applications.

### 4.1. Metal-Free Active Site Design

Currently, different types of metal-free COFs have been successfully prepared and applied in the electrosynthesis of H_2_O_2_ from 2e^−^ ORR (Table 1).

**Figure 4:**
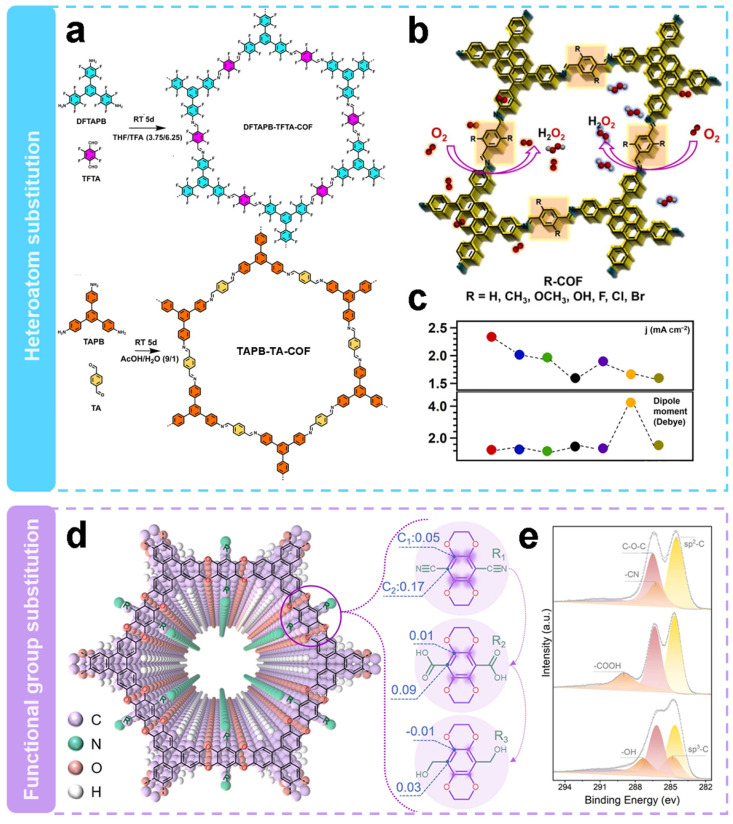
Regulation of side-chain substitution. (**a**,**b**) Schematic representation of substitution via heteroatoms [58]. (**c**) Activity and dipole moment images of H-COF (black curve), Br-COF (red curve), Cl-COF (blue curve), F-COF (green curve), OH-COF (purple curve), OCH_3_-COF (orange curve), and CH_3_-COF (yellow curve) [66]. (**d**) Schematic representation of substitution through functional groups. (**e**) XPS spectra of C1s [65].

Substituting side chains with different heteroatoms is a strategy to induce active sites in metal-free electrocatalysis. Segura et al. [58] integrated fluorine atoms in a defined sp^2^ backbone to enhance the H_2_O_2_ selectivity. Then the structure–performance relationships were revealed. Highly fluorinated COFs (DFTAPB-TFTA-COF) and homo-structurally non-fluorinated COFs (TAPB-TA-COF) were prepared at room temperature by Schiff base condensation reactions as shown in Figure 4a. The as-obtained COFs show significant differences in their electron pathways during ORR. The electron transfer number of DFTAPB-TFTA-COF was 2.1, which shows it predominantly undergoes the two-electron pathway, whereas the electron transfer number of TAPB-TA-COF was 3.2, which shows the existence of mixed pathway. It is worth noting that the fluorine substitution on DFTAPB-TFTA-COF improves the selectivity of the electrochemical process for the H_2_O_2_ generation. The H_2_O_2_ selectivity of fluorinated COFs was as high as 96.25% with a Faraday efficiency of 71.1%, which was 2.15 and 2.48 times higher than that of hydrogenated COFs, respectively. The H_2_O_2_ yield rate of DFTAPB-TFTA-COF was 253 mmol g_catlayst_^−1^ h^−1^. The higher electrocatalytic selectivity of fluorinated COFs as compared with that of hydrogenated COFs could be attributed to the polarized sp^2^ C–F bond, which served as active sites for O_2_ adsorption.

Apparently, the electronic characteristics of COFs have great influence on the electrocatalytic activity of 2e^−^ ORR. As shown in Figure 4b, Jiang’s team [66] investigated the effect of substituents on the catalytic performance of 2e^−^ ORR by modulating their electronic properties. A basic COF (H-COF) without substituents was synthesized by solvothermal method, and by changing the electronic properties of the substituents in the side chains, R-COFs (R = CH_3_, OCH_3_, OH, F, Cl or Br) with similar topological capabilities but different band gaps and polarities were obtained. The electron-withdrawing substituents help to achieve higher 2e^−^ ORR activity. As shown in Figure 4c, the order of catalytic activity follows Br-COF > Cl-COF > OH-COF > F-COF > CH_3_-COF > CH_3_O-COF > H-COF. The H_2_O_2_ selectivity of H-COF was 76.9–73.3%, whereas the selectivity of Br-COF, Cl-COF, and F-COF was 85.4–85.5%, 84.4–82.4%, and 81.2–78.1%, respectively. The selectivity of H_2_O_2_ was significantly enhanced by the introduction of electron-withdrawing substituents. In contrast, the H_2_O_2_ selectivity of COFs with electron-donating substituents were similar or slightly lower compared to that of H-COF. At a potential of 0.5 V vs. RHE, the calculated mass activity and TOF values of Br-COF are 32.0 A g^−1^ and 0.2498 s^−1^, respectively, which are 112% and 174% higher than those of the unmodified H-COF. The experimental and theoretical results indicate that the catalytic activity mainly depends on the reduction ability of COFs. Moreover, the introduction of Br atoms in the linker improves the activity due to the easy formation of OOH* intermediates.

Wang’s team [65] also modulated the charge states of neighboring carbon atoms by introducing functional groups with different electron affinities in the COFs. As can be seen in Figure 4d, three COFs (COF-CN, -COOH or -CH_2_OH) were designed with the same backbone and active site density. The XPS patterns of the C1s in Figure 4e show characteristic peaks of -CN, -COOH, and -OH, demonstrating the successful introduction of different functional groups. The COF-CN with the strongest electron withdrawing groups, exhibited the best electrocatalytic activity, including the most positive onset potential (0.72 V vs. RHE) and the largest ring and disk currents (2.12 and 1.96 mA cm^−2^, respectively). The H_2_O_2_ selectivity of these COFs were in the order of COF-CN > COF-COOH > COF-CH_2_OH. COF-CN had the highest H_2_O_2_ selectivity of 97.2%, and the electron transfer number of 2.06. Combined with the theoretical calculations, the results indicated that the strong electron-withdrawing ability of -CN contributed to the generation of more positive charges in the active site. This favors the adsorption of the intermediate product OOH*, which promotes reaction kinetics and selectivity.

**Figure 5:**
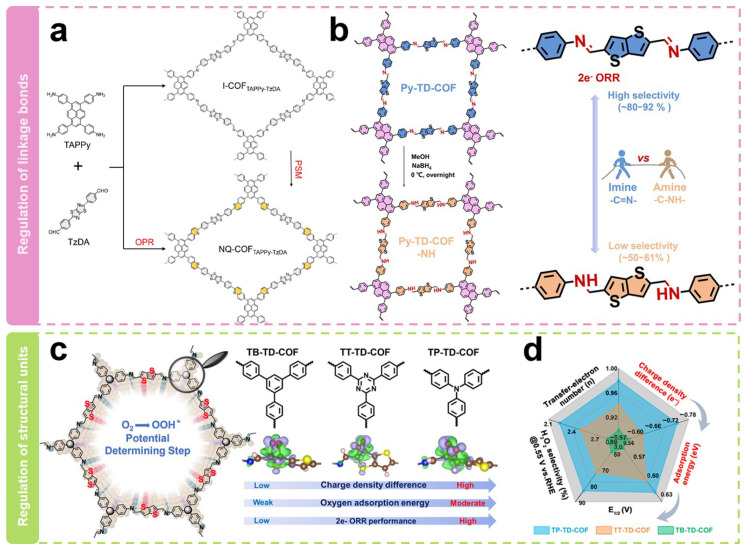
Regulation of backbone structure. (**a**,**b**) Schematic representation of the regulation of link bonds [62,64]. (**c**) Schematic diagram of the regulation of structural units. (**d**) Relationship diagram of structure activity [63].

Modulating the linkage to alter the geometric shape and electronic structure of active sites is a noteworthy strategy. As shown in Figure 5a, Xiang et al. [64] synthesized two quinoline-linked thiazole COFs (NQ-COF_TAPPy-TzDA_-PSM and NQ-COF_TAPPy-TzDA_-OPR) via the post-synthesis modification route (PSM) and one-pot reaction route (OPR). Among them, the PSM route converted the imine-linked bonds in I-COF_TAPPy-TzDA_ to unsubstituted quinolines. The obtained COFs delivered electron transfer numbers of 2.61–2.77, H_2_O_2_ selectivity of 61–69%, and Faraday efficiencies of 66.7% in the neutral condition of 0.1 M Na_2_SO_4_, which were significantly higher than those of imine-linked COFs (electron transfer numbers of 3.2, selectivity of 34–41%, and Faraday efficiency of 39.5%). Analysis of the electronic structure shows that the improved 2e^−^ ORR performance after the structural transformation is attributed to the in-plane π-electron departure from the domain. The electron migration induces charge redistribution around the active site, which optimizes the adsorption of oxygen intermediates. Our research group [62] has recently reported two COFs with different linkages toward the electrocatalytic production of H_2_O_2_ (Figure 5b). We firstly constructed a typical imine-linked COF (Py-TD-COF). COFs linked by amines (Py-TD-COF-NH) were then synthesized by a simple direct reduction method. Their electrocatalytic ORR performance was then investigated. It was found that there were only minor differences between their onset potentials (E_0_), half-wave potentials (E_1/2_), and diffusion-limited current densities. However, a significant difference in their ring current densities was observed, which suggested their diverse H_2_O_2_ selectivity. Py-TD-COF linked by imine exhibited typical donor–acceptor properties with electron transfer numbers of 2.2–2.5 and H_2_O_2_ selectivity of 80–92%, suggesting that it predominantly undergoes the 2e^−^ pathway during the ORR process. In contrast, Py-TD-COF-NH linked by amine showed relatively higher electron transfer numbers of 2.8–3.0 and lower H_2_O_2_ selectivity of 50–61%. The relatively good selectivity of the imine bond is due to the relatively strong interaction, which stabilizes the critical OOH* intermediate. Simultaneously, the hydrogen bonding interactions within the amine chain elongate the O–O bond of O_2_* and the crucial intermediate OOH*, weakening the O–O bond.

Modulating different structural units can also regulate the selectivity of H_2_O_2_. As shown in Figure 5c, structural units with different tridentate nodes with unique electronic configurations have recently been selected by our group [63] to construct COFs with different electronic structures (TB-TD-COF, TT-TD-COF, and TP-TD-COF). Theoretical calculations showed that the true active sites are carbon atoms adjacent to thiophene-S. Their adsorption capacity for O_2_ molecules can be precisely regulated by adjusting the charge density difference between the active sites and O_2_. Furthermore, the adsorption energy of O_2_ molecules on the true active sites is positively correlated with the 2e^−^ ORR performance. As shown in Figure 5d, the H_2_O_2_ selectivity on TP-TD-COF was 85% with an average electron transfer number of 2.24 at a potential of 0.55 V vs. RHE. At the same time, the H_2_O_2_ selectivities on TT-TD-COF and TB-TD-COF were 71% and 62%, with an average electron transfer number of 2.52 and 2.86, respectively. The superior selectivity of TP-TD-COF could be attributed to the fact that the active unit on TP-TD-COF had the richest electron density and the strongest electron-donating ability to O_2_ molecules. The H_2_O_2_ yield rate catalyzed by TP-TD-COF reached 158 mmol g^−1^_catalyst_ h^−1^, which may have potential applications.

**Figure 6:**
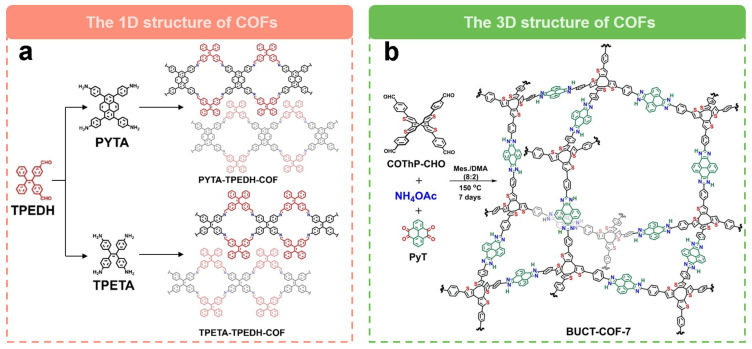
Schematic representation of dimensional regulation of COFs. (**a**) Schematic of the preparation of 1D COFs [61]. (**b**) Schematic of the preparation of 3D COFs [60].

The unique dimensional design of COFs to tune their electrocatalytic performance for the generation of H_2_O_2_ is also a powerful strategy. A recent study by Hu et al. [61] used a one-dimensional COF (PYTA-TPEDH-COF) as a 2e^−^ ORR electrocatalyst. In order to elucidate the effects of different COF dimensions on 2e^−^ ORR, they synthesized a two-dimensional COF (PYTA-TPETH-COF) with the same catalytic sites for comparison. They found that the electrochemically active surface area (ECSA) of PYTA-TPEDH-COF was 4.8 times higher than that of the 2D COF, which confirms that the one-dimensional topology provides more active surfaces and exposed catalytic sites in the catalytic process. Meanwhile, the imine bond can electrocatalyze the ORR via the 2e^−^ pathway with high activity and selectivity. The one-dimensional COF, PYTA-TPEDH-COF, has excellent 2e^−^ ORR activity with higher H_2_O_2_ selectivity (85.8–82.0%) and lower electron transfer number (2.28–2.36) compared to a two-dimensional COF, PYTA-TPETH-COF (H_2_O_2_ selectivity are 72.9–69.0% and electron transfer number are 2.54–2.62). Recently, Wang’s group [60] reported a fully conjugated 3D COFs for the efficient electrochemical synthesis of H_2_O_2_. As shown in Figure 6b, they synthesized a 3D COF (BUCT-COF-7) with a heterocyclic imidazolium ring as the linker via a one-pot multicomponent method. Due to the robust structure with π-conjugation and abundant heteroatom content, BUCT-COF-7 showed excellent electrocatalytic activity for 2e^−^ ORR. After mixing with single-walled carbon nanotubes, the COF composites showed good ORR activity with an onset potential of 0.82 V vs. RHE, a H_2_O_2_ selectivity close to 83.4% at 0.66 V vs. RHE, and an electron transfer number of 2.41. The H_2_O_2_ yield rate was as high as 326.9 mmol g_catlayst_^−1^ h^−1^ with good stability.

### 4.2. Metalated Active Sites Design

Besides the metal-free active sites, the selection of suitable ligands to chelate metallic species in the COF backbone for electrocatalysis is also a commonly used strategy. Typical COFs with metalated active sites as electrocatalysts for 2e^−^ ORR are summarized in Table 2. The introduction of metal in the COFs can lead to improved selectivity. Zeng and his coauthors [53] obtained MgP-DHTA-COFs using porphyrin as a chelating ligand. The designed MgP-DHTA-COFs showed a H_2_O_2_ selectivity up to 96%, while the selectivity of the uncoordinated COF (H_2_P-DHTA-COF) was only 76.1%. It was experimentally and theoretically demonstrated that the pyrrole N-immobilized Mg ions as metallic sites could promote the reactivity of COFs and enhance the adsorption capacity for OOH*. Jiang’s team [59] introduced the noble metal Pt into the COF. They designed a novel COF (TP-TTA-COF) consisting of alkyne and chelating groups, which was obtained by immobilizing highly dispersed platinum in a one-dimensional porous channel (PtCl-COF). After Pt-atom embedding, PtCl-COF exhibited enhanced H_2_O_2_ selectivity from 81.6% to 87.2%, and an electron transfer number from 2.37 to 2.26. Theoretical calculations suggest that the strong Pt-carrier interaction promotes the formation of OOH*, which improves the selectivity and activity of 2e^−^ ORR. In addition, the electronic structure of the active site can also be effectively modulated by the selection of the metallic center, thus modulating the ORR activity of the catalyst.

**Figure 7:**
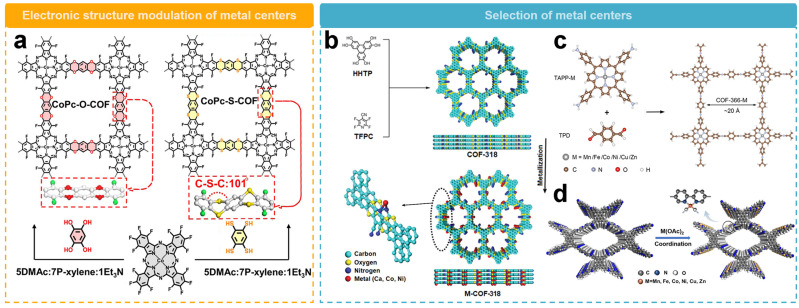
Schematic representation of the activity regulation of metallic sites. (**a**) Schematic diagram of regulation of COFs through linker units [52]. (**b**) Schematic diagram illustrating the regulation of metallic centers by dioxin-conjugated COF [55]. (**c**) Schematic diagram illustrating the regulation of metallic centers by COFs rich in conjugated porphyrin groups [54]. (**d**) Schematic diagram illustrating the regulation of metallic centers by COFs rich in bipyridine groups [56].

Modulating the electronic structure of the metallic center by changing COF linkers is one of the ways to optimize the 2e^−^ ORR performance. Jiang’s team [52] recently prepared a porous dithiobiphenyl-based COF (CoPc-S-COF), along with a conventional dioxin-linked two-dimensional COF (CoPc-O-COF) (Figure 7a). The CoPc-S-COF has a high electrocatalytic ORR activity with an onset potential of 0.81 V, which is higher than that of CoPc-O-COF (0.78 V). The H_2_O_2_ selectivity of CoPc-S-COF is over 90% with an electron transfer number of 2.0–2.2, superior to CoPc-O-COF in the same potential range. Compared with CoPc-O-COF with π-stacking model, CoPc-S-COF is bent along the C–S–C bridge with undulating layer stacking structure, which exposes more cobalt centers. Also, the electron-donating effect of S atoms almost benefit the activity of Co sites and weaken H_2_O_2_ decomposition, resulting in its higher H_2_O_2_ selectivity.

The strategy of regulating the activity of metal sites by adjusting the metal center has been wildly used. Zeng’s team [55] produced a series of electrocatalysts (M-COF-318, M = Ca, Co, Ni, Fe, or Cu) for H_2_O_2_ synthesis by utilizing dioxin-conjugated COFs as a template for the precise construction of different metal-N_2_ sites along the porous walls (Figure 7b). By employing various metallic centers, the activity and selectivity of the catalysts can be effectively tuned. In the potential range of 0.2 to 0.6 V vs. RHE, Ca-COF-318 could deliver a H_2_O_2_ selectivity over 95% and a Faraday efficiency of 91%. In comparison, the H_2_O_2_ selectivity of Co or Ni sites were 20% and 60%, respectively, in the same potential range. In addition, the turnover frequency (TOF) of the Ca–N_2_ site was measured to be 11.63 e^−1^ site^−1^ s^−1^, which is 58 and 20 times higher than those of the Co–N_2_ and Ni–N_2_ sites. Theoretical calculations further indicated that the Ca site has a weaker binding affinity for OOH* than the Co or Ni sites, thus facilitating the desorption of OOH* and thus catalyzing the 2e^−^ ORR efficiently. In addition, the long-term stability measurements showed that the activity and selectivity of Ca-COF-318 remained good even after 10 h. The H_2_O_2_ generation rate of Ca-COF-318 was 453 mmol g_catalyst_^−1^ h^−1^.

The method of regulating the metal center is applicable to conjugated porphyrin groups in COF materials. In Figure 7c, Chen’s team [54] synthesized a series of metal–nitrogen–carbon (M–N–C) single-atom catalysts based on conjugated porphyrin-based COFs. A covalent organic framework with the same metal coordination environment was used as a model catalyst (COF-366-M) and the catalytic activities and selectivity of different metallic centers (Mn, Fe, Co, Ni, Cu, and Zn) were compared for the production of H_2_O_2_ in alkaline, neutral, and acidic electrolytes. All tested COF-366-M catalysts exhibited higher disk current densities and more positive onset potentials compared to COF-366, suggesting that the metallic atoms in COF-366-M are active catalytic sites. The overall H_2_O_2_ selectivities depend on the metallic species with a trend of COF-366-Co > Ni > Cu > Fe > Zn > Mn. COF-366-Co is the most suitable electrocatalyst with a H_2_O_2_ selectivity up to 91% and the Faraday efficiency up to 84% in 0.1 M KOH. The yield rate is 909 mmol g_catalyst_^−1^ h^−1^, demonstrating its superior performance towards 2e^−^ ORR. A combination of experiments and calculations revealed that the initial O_2_ adsorption and stability of OOH* intermediates jointly determine the activity and selectivity of H_2_O_2_ generation. The difference in binding energies (E_O2*_-E_OOH*_) of O_2_* and OOH* intermediates on a single metallic center is a reliable thermodynamic descriptor for predicting the catalytic activity of metallic centers.

The selectivity of 2e^−^ ORR can also be modulated by the metal centers in pyridine-based COFs. As shown in Figure 7d, our group [56] developed a series of single-atom electrocatalysts anchored on bipyridine-rich COF (Py-Bpy-COF-M, M = Mn, Fe, Co, Ni, Cu, or Zn) for selective 2e^−^ ORR. Compared to Py-Bpy-COF, all Py-Bpy-COF-M catalysts evaluated in 0.1 M KOH exhibited higher current densities and more positive onset potentials. The order of 2e^−^ ORR performance of different metallic centers follow the order, Py-Bpy-COF-Zn > Ni > Fe > Co > Cu > Mn. Notably, Py-Bpy-COF-Zn maintained a high H_2_O_2_ selectivity of more than 95.0% in the potential range of 0.3–0.6V vs. RHE, and the highest selectivity reached up to 99.1%. Its Faraday efficiency exceeded 90% over a wide potential range, which was significantly higher than that of other active metallic centers at the same potential. In addition, Py-Bpy-COF-Zn also exhibits excellent stability, maintaining disk and ring current densities with only slight fluctuations after 10 h of continuous operation at 0.55V vs. RHE. Meanwhile, the H_2_O_2_ selectivity can be maintained at about 94.0% throughout the operation. The high 2e^−^ ORR performance exhibited by Py-Bpy-COF-Zn can be attributed to the high OOH* dissociation barrier as indicated by theoretical calculations.

## 5. Conclusions and Perspective

COFs have attracted much attention in the field of electrocatalytic 2e^−^ ORR because of their precise pores and compositions, stable conjugated structures, and easily functionalized ordered framework structures. Through rational selection of building blocks, COFs are capable of realizing pre-designed compositions and functions. Through the introduction of metallic species, heteroatom substitutions, or functional groups, the electronic structure and the catalytic activity of COFs can be effectively modulated. By designing different dimensional COFs, the active surface and active site distribution could be changed. All of them can guide the rational design of high-performance 2e^−^ ORR electrocatalysts. This review introduces the design principles of COFs and their specific applications in 2e^−^ ORR. Using the active site as a medium, the design strategies of COFs with metal-free active sites and metalated active sites are highlighted. Many COFs have demonstrated excellent 2e^−^ ORR performance in previous studies, but it needs to be clearly pointed out that the designed COF-based catalysts still have many limitations in practical applications, which need to be further explored and investigated.

**a. The further improvement of COF-based 2e^−^ ORR electrocatalysts.** The typical low electrical conductivity and relatively low durability of COFs make them somewhat limited in practical applications. The optimization and enhancement of the electrical conductivity of COFs will be of great significance to the promotion of the COFs’ practical application toward 2e^−^ ORR. Moreover, it is crucial to developing structure-specific COFs catalysts for targeted catalysis of ORR. New strategies, such as machine learning, may be used to screen and optimize the structure of COFs, which will lead to a more accurate and efficient development of COF-based 2e^−^ ORR electrocatalysts. In addition, the development of COF scale-up synthesis and processing should be explored in the future to realize the practical applications of COFs.

**b. Precise structure-activity relationship of COF-based 2e^−^ ORR electrocatalysts.** The reaction process and mechanism of electrocatalytic ORR in COF materials need to be further explored. To clearly understand the structure of COF-based catalysts and the changes in intermediates during the electrocatalysis, more advanced characterization methods and theoretical computational tools need to be developed. The precise structure-activity relationship of COF-based 2e^−^ ORR electrocatalysts is crucial to guiding the development of efficient and novel COF-based 2e^−^ ORR catalysts. With the more clearly revealed catalytic mechanism and behaviors of COF-catalyzed 2e^−^ ORR process, the development of highly efficient novel COF-based 2e^−^ ORR catalysts would be accelerated.

**c. The possible industrial applications of COF-based 2e^−^ ORR electrocatalysts.** The development of COF-based 2e^−^ ORR electrocatalysts from the lab to the market requires overcoming key challenges. Scalable, cost-effective production methods must be developed to ensure consistent quality and performance. Integration into existing industrial systems and ensuring durability and stability under operational conditions are crucial for practical application. The unique properties and tunability of COFs make them highly promising in energy storage, environmental remediation, and other fields, driving sustainable industrial processes and innovations.

## Figures and Tables

**Table 1:** Typical COFs with metal-free active sites as electrocatalysts for 2e^−^ ORR.

Electrocatalysts	Electrolyte	n	H_2_O_2_ Selectivity (%)	FE (%)	E_0_/V (vs. RHE)	E_1/2_/V (vs. RHE)	J_lim_/(mA cm^−2^)	Ref.
cCTN:Cl^−^	0.1 M KOH	2.2	85.3	N/A	0.75	~0.60	N/A	[57]
DFTAPB-TFTA-COF	0.1 M NaOH	2.1	96.25	71.1	0.698	~0.60	1.70	[58]
TP-TTA-COF	0.1 M KOH	2.58–2.68	66.0–70.9	N/A	0.622	~0.57	1.32	[59]
BUCT-COF-7/CNT	0.1 M KOH	2.41	83.4	~80	0.82	~0.71	N/A	[60]
PYTA-TPEDH-COF	0.1 M KOH	2.28–2.36	82–85.8	~80	0.69	~0.60	2.14	[61]
TPETA-TPEDH-COF		2.40–2.50	75.2–79.8	N/A	0.69	~0.60	1.90	
PYTA-TPETH-COF		2.54–2.62	69–72.9	N/A	0.69	~0.60	2.13	
Py-TD-COF	0.1 M KOH	2.2–2.5	80–92	N/A	0.834	0.698	2.898	[62]
Py-TD-COF-NH		2.8–3.0	50–61	N/A	0.829	0.693	2.891	
TB-TD-COF	0.1 M KOH	2.86	50.9–67.4	N/A	0.71	0.55	N/A	[63]
TP-TD-COF		2.24	81.9–86.2	N/A	0.76	0.62	N/A	
TT-TD-COF		2.52	68.9–81.8	N/A	0.74	0.60	N/A	
NQ-COF_TAPPy-TzDA_-OPR	0.1 M Na_2_SO_4_	2.61–2.77	61–69	66.7	~0.12	~−0.2	N/A	[64]
I-COF_TAPPy-TzDA_		~3.2	34–41	39.5	~0.02	~−0.3	N/A	
NQ-COF_TAPPy-TzDA_-PSM		~2.8	48–57	61.3	~−0.02	~−0.25	N/A	
COF-CN	0.1 M KOH	2.06	97.2	N/A	0.72	~0.64	N/A	[65]
COF-CH_2_OH		~2.3	~82%	N/A	0.68	~0.60	N/A	
COF-COOH		~2.2	~89	N/A	~0.70	~0.62	N/A	
Br-COF	0.1 M KOH	2.26–2.29	85.4–85.5	80.6	0.70	0.61	2.34	[66]
Cl-COF		~2.31	82.4–84.4	N/A	0.67	0.60	1.99	
OCH_3_-COF		~2.33	80.1–81.9	N/A	0.66	0.59	1.67	
COF-366	0.1 M KOH	2.4	78	64	~0.60	~0.51	N/A	[54]
	0.1 M PBS	2.8	58	41	~0.50	~0.31	N/A	
	0.1 M ABS	2.9	53	36	~0.25	~0.13	N/A	
Py-Bpy-COF	0.1 M KOH	2.69	63.5	N/A	~0.67	~0.60	N/A	[56]
H_2_P-DHTA-COF	0.1 M KOH	2.45–2.49	76.1	N/A	0.67	0.57	1.4	[53]

“N/A” means not available.

**Table 2:** Typical COFs with metalated active sites as electrocatalysts for 2e^−^ ORR.

Electrocatalysts	Electrolyte	n	H_2_O_2_ Selectivity (%)	FE (%)	E_0_/V (vs. RHE)	E_1/2_/V (vs. RHE)	J_lim_/(mA cm^−2^)	Ref.
MgP-DHTA-COF	0.1 M KOH	2.11–2.15	96	90.6	0.68	0.60	2.0	[53]
PtCl-COF	0.1 M KOH	2.26–2.37	81.6–87.2	N/A	0.675	~0.58	1.83	[59]
CoPc-S-COF	0.1 M KOH	2.0–2.2	~94	~95	0.81	~0.72	N/A	[52]
CoPc-O-COF		~2.3	~88	~90	0.78	~0.68	N/A	
Py-Bpy-COF-Zn	0.1 M KOH	2.06	99.1	N/A	~0.75	~0.65	N/A	[56]
Py-Bpy-COF-Fe		2.61	45.1	N/A	~0.79	~0.66	N/A	
Py-Bpy-COF-Ni		2.29	79.8	N/A	~0.75	~0.66	N/A	
Ca-COF-318	0.1 M KOH 0.1 M KOH 0.1 M KOH	2.1	94–95	91	0.75	0.61	2.80	[55]
COF-366-Co	0.1 M KOH	2.2	91	84	~0.67	~0.58	N/A	[54]
COF-366-Ni		2.3	86	75	~0.68	~0.60	N/A	
COF-366-Cu		2.5	76	61	~0.64	~0.55	N/A	
COF-366-Co	0.1 M PBS	2.3	86	75	~0.60	~0.20	N/A	
COF-366-Ni		2.4	80	67	~0.60	~0.20	N/A	
COF-366-Cu		2.5	75	60	~0.50	~0.20	N/A	
COF-366-Co	0.1 M ABS	2.3	87	77	~0.43	~0.19	N/A	
COF-366-Ni		2.4	79	65	~0.42	~0.19	N/A	
COF-366-Cu		2.7	65	48	~0.25	~0.13	N/A	

“N/A” means not available.

## Data Availability

No new data were created or analyzed in this study. Data sharing is not applicable to this article.
